# Supplementary material for: Revealing Ancient Wheat Phylogenetic Diversity: Machine Learning and Logistic Regression Identify Triticum sphaerococcum in Bronze Age Iberia
Source: Genes (Basel). 2025 Dec 9;16(12):1477. doi: 10.3390/genes16121477 (PMC12733154; doi:10.3390/genes16121477)
Supplement: Supplementary file 1 [file genes-16-01477-s001.zip › Supplementary Table S1.pdf]

Supplementary Table S1. ^{14}C dates from Punta de Los Gavilanes site (Mazarrón. Murcia. Spain)

(Columns include: Phase. Sample nature. Sample code and Laboratory code; BP date, cal y. BP median probability, cal. y. BCE/CE probability ranges (at 1σ and 2σ) and 2σ median probability) (Gavilanes Project Archive).

Phase	Sample	Sample code	Lab. code	BP date	Cal. y. BP date. median probability	Cal years BCE/CE date * (1σ probability ranges)	Cal years BCE/CE date * (2σ probability ranges)	Median 2σ probability
GV-IVa1 TSM	burnt animal bone	GV-3321-22.1	KIA-43336	3855±30	4280	2460 BCE (20.9%) 2370 BCE. 2350 BCE (39.2%) 2280 BCE. 2250 BCE (6.8%) 2230 BCE. 2220 BCE (1.3%) 2210 BCE	2460 BCE (79.9%) 2270 BCE. 2260 BCE (15.5%) 2200 BCE	-2330
GV-IVa1 TM	seeds	GV-2076-23.1	KIA-43323	3760±30	4123	2280 BCE (8.3%) 2250 BCE. 2210 BCE (59.9%) 2130 BCE	2290 BCE (82.8%) 2120 BCE. 2090 BCE (12.6%) 2040 BCE	-2174
GV-IVa1 TM	charcoal	GV-2033-25.1	KIA-32355	3730±30	4080	2200 BCE (22.6%) 2160 BCE. 2150 BCE (15.5%) 2120 BCE. 2090 BCE (30.1%) 2040 BCE	2210 BCE (95.4%) 2030 BCE	-2130
GV-IVa1 TM	seeds	GV-2077-23.1	KIA-43324	3700±30	4038	2140 BCE (68.2%) 2030 BCE	2200 BCE (8.8%) 2160 BCE. 2150 BCE (84.3%) 2010 BCE. 2000 BCE (2.4%) 1980 BCE	-2089
GV-IVa1 TM	charcoal	GV-2080-25.1	KIA-43325	3700±35	4038	2140 BCE (68.2%) 2030 BCE	2200 BCE (91.3%) 2010 BCE. 2000 BCE (4.1%) 1970 BCE	-2089
GV-IVa2 TM	charcoal	GV-2074-25.1	KIA-43322	3660±30	3984	2130 BCE (26.0%) 2080 BCE. 2050 BCE (42.2%) 1970 BCE	2140 BCE (95.4%) 1940 BCE	-2035
GV-IVa2 TM	human bone	GV-1581-22.1	KIA-336021	3660±30	3984	2130 BCE (26.0%) 2080 BCE. 2050 BCE (42.2%) 1970 BCE	2140 BCE (95.4%) 1940 BCE	-2035
GV-IVa2 TS	charcoal	GV-1527-25.1	KIA-32356	3650±30	3968	2120 BCE (12.3%) 2090 BCE. 2040 BCE (55.9%) 1960 BCE	2140 BCE (95.4%) 1930 BCE	-2019
GV-IVa2 TS	charcoal	GV-1818-25.1	KIA-37601	3645±35	3962	2120 BCE (10.5%) 2090 BCE. 2040 BCE (57.7%) 1950 BCE	2140 BCE (95.4%) 1910 BCE	-2013
GV-IVa2 TS	seeds	GV-1818-23.1	KIA-37597	3620±50	3935	2110 BCE (2.3%) 2100 BCE. 2040 BCE (65.9%) 1900 BCE	2140 BCE (95.4%) 1870 BCE	-1986
GV-IVa2 TS	seeds & charcoal	Average GV-1818	KIA-37601 &KIA-37597	3637±29		2035 BCE (68.2%) 1950 BCE	2140 BCE (13.0%) 2080 BCE. 2060 BCE (82.4%) 1910 BCE	-1986
GV-IVa2 TS	seeds	GV-1808-23.1	KIA-37593	3625±35	3937	2035 BCE (68.2%) 1935 BCE	2130 BCE (9.5%) 2080 BCE. 2050 BCE (85.9%) 1890 BCE	-1988
GV-IVa2 TM	charcoal	GV-1306-C.1	KIA-19484	3605±30	3903	2020 BCE (14.8%) 1990 BCE. 1980 BCE (50.9%) 1910 BCE. 1900 BCE (2.5%) 1890 BCE	2040 BCE (95.4%) 1880 BCE	-1964
GV-IVa2 TM	charcoal	GV-1306-C.2	KIA-19213	3595±30	3900	2010 BCE (0.09%) 2000 BCE. 1980 BCE (0.91%) 1890 BCE	2040 BCE (0.97%) 1880 BCE. 1840 BCE (0.03%) 1820 BCE	-1951
GV-IVa2 TS	seeds	GV-1799-23.1	KIA-37596	3590±45	3896	2020 BCE (11.6%) 1990 BCE. 1980 BCE (56.6%) 1880 BCE	2130 BCE (3.3%) 2090 BCE. 2050 BCE (84.1%) 1860 BCE. 1850 BCE (8.0%) 1770BCE	-1947
GV-IVb TM	charcoal	GV-2013-25.1	KIA-32353	3560±25	3860	1945 BCE (68.2%) 1880 BCE	2020 BCE (1.6%) 1990 BCE. 1980 BCE (82.3%) 1870 BCE. 1850 BCE (7.1%) 1810 BCE. 1800 BCE (4.3%) 1770 BCE	-1911

Phase	Sample	Sample code	Lab. code	BP date	Cal. y. BP date. median probability	Cal years BCE/CE date * (1σ probability ranges)	Cal years BCE/CE date * (2σ probability ranges)	Median 2σ probability
GV-IVb TS	seeds	GV-1776-23.1	KIA-37594	3575±35	3877	1975 BCE (68.2%) 1880 BCE	2030 BCE (86.9%) 1870 BCE. 1850 BCE (5.2%) 1810 BCE. 1800 BCE (3.4%) 1770 BCE	-1928
GV-IVb TS	charcoal	GV-1776-25.1	KIA-37591	3550±35	3844	1950 BCE (51.4%) 1870 BCE. 1850 BCE (9.9%) 1820 BCE. 1800 BCE (6.9%) 1780 BCE	2020 BCE (1.9%) 1990 BCE. 1980 BCE (93.5%) 1770 BCE	-1895
GV-IVb TS	seeds & charcoal	Average GV-1776	KIA-37594KIA-37591	3563±25		1945 BCE (68.2%) 1885 BCE.	2020 BCE (1.9%) 1990 BCE. 1980 BCE (85.0%) 1870 BCE. 1850 BCE (5.4%) 1810 BCE. 1800 BCE (3.0%) 1780 BCE	-1925
GV-IVb TM	charcoal	GV-2052-25.1	KIA-43320	3530±30	3796	1920 BCE (30.3%) 1870 BCE. 1850 BCE (37.9%) 1770 BCE	1950 BCE (95.4%) 1750 BCE	-1847
GV-IVb TM	charcoal	GV-2053-25.1	KIA-43321	3535±35	3815	1930 BCE (34.9%) 1870 BCE. 1850 BCE (33.3%) 1770 BCE	1960 BCE (95.4%) 1750 BCE	-1866
GV-IVb TM	human bone	GV-1649-22.1	KIA-33602	3515±30	3781	1890 BCE (15.5%) 1860 BCE. 1850 BCE (52.7%) 1770 BCE	1920 BCE (95.4%) 1750 BCE	-1832
GV-IVb TM	charcoal inner rings	GV-2120-25.1	RICH-23290	3494±39	3767	1880 BCE (68.2%) 1760 BCE	1920 BCE (91.3%) 1730 BCE. 1720 BCE (4.1%) 1690 BCE	-1818
GV-IVb TM	charcoal outer rings	GV-2120-25.2	RICH-23289	3570±38	3871	2010 BCE (3.1%) 2000 BCE. 1980 BCE (65.1%) 1880 BCE	2030 BCE (82.0%) 1860 BCE. 1850 BCE (13.4%) 1770 BCE	-1922
GV-IVb TM	esparto fibers	GV-2120-25.3	RICH-23284	3466±35	3743	1880 BCE (22.1%) 1840 BCE. 1830 BCE (40.9%) 1740 BCE. 1710 BCE (5.2%) 1690 BCE	1890 BCE (95.4%) 1690 BCE	-1794
GV-IVb TM	esparto fibers	GV-1451-25.1	KIA-43319	3485±30	3762	1880 BCE (26.6%) 1840 BCE. 1830 BCE (41.6%) 1750 BCE	1890 BCE (93.8%) 1730 BCE. 1710 BCE (1.6%) 1690 BCE	-1813
GV-IVb TM	seeds	GV-1451-23.1	KIA-40419	3475±30	3755	1880 BCE (25.5%) 1840 BCE. 1830 BCE (42.7%) 1740 BCE	1890 BCE (91.3%) 1730 BCE. 1720 BCE (4.1%) 1690 BCE	-1806
GV-IVb TS	human bone	GV-1710-22.1	KIA-40417	3535±30	3817	1930 BCE (36.1%) 1870 BCE. 1850 BCE (18.7%) 1810 BCE. 1800 BCE (13.5%) 1770 BCE	1950 BCE (95.4%)1750 BCE	-1868
GV-IVb TS	seeds rem.	GV-1719-23.1	KIA-40426	3425±30	3673	1770 BCE (68.2%) 1685 BCE	1880 BCE (8.6%) 1840 BCE. 1820 BCE (3.0%) 1790 BCE. 1780 BCE (83.8%) 1630 BCE	-1724
GV-IVb TS	seeds rem.	GV-1688-23.1	KIA-40421	3445±35	3705	1870 BCE (13.9%) 1840 BCE. 1820 BCE (3.8 %) 1800 BCE. 1780 BCE (50.5%) 1690 BCE	1890 BCE (95.4%) 1660 BCE	-1756
GV-IVb TSM	charcoal	GV-3197-25.1	KIA-37595	3485±30	3762	1880 BCE (68.2%) 1750 BCE	1920 BCE (90.2%) 1730 BCE. 1720 BCE (5.2%) 1690BCE	-1813
GV-IVb TSM	esparto fibers	GV-3145-25.1	KIA-37602	3445±30	3703	1870 BCE (13.3%) 1840 BCE. 1810 BCE (1.8%) 1800 BCE. 1780 BCE (53.1%) 1690 BCE	1880 BCE (95.4%) 1680 BCE	-1754
GV-IVb TSM	charcoal	GV-3180-25.1	KIA-37599	3445±30	3703	1870 BCE (13.3%) 1840 BCE. 1810 BCE (1.8%) 1800 BCE. 1780 BCE (53.1%) 1690 BCE	1880 BCE (95.4%) 1680 BCE	-1754
GV-IVb TSM	charcoal	GV-3194-25.1	KIA-37598	3430±30	3680	1770 BCE (68.2%) 1680 BCE	1880 BCE (11.1%) 1840 BCE. 1830 BCE (84.3%) 1630 BCE	-1731
GV-IVb TSM	charcoal	GV-3244-25.1	KIA-37592	3430±30	3680	1770 BCE (68.2%) 1680 BCE	1880 BCE (11.1%) 1840 BCE. 1830 BCE (84.3%) 1630 BCE	-1731

Phase	Sample	Sample code	Lab. code	BP date	Cal. y. BP date. median probability	Cal years BCE/CE date * (1σ probability ranges)	Cal years BCE/CE date * (2σ probability ranges)	Median 2σ probability
GV-IVb TSM	charcoal	GV-3103-25.1	KIA-37600	3410±30	3659	1750 BCE (68.2%) 1665 BCE	1870 BCE (2.7%) 1840 BCE. 1780 BCE (92.7%) 1620 BCE	-1710
GV-IVc TS	seeds	GV-1743-23.1	KIA-32366	3385±35	3629	1740 BCE (19.6%) 1710 BCE. 1700 BCE (48.6%) 1630 BCE	1770 BCE (92.3%) 1600 BCE. 1580 BCE (3.1%) 1530 BCE	-1680
GV-IVc TS	seeds rem.	GV-1711-23.1	KIA-40418	3390±30	3634	1740 BCE (26.2%) 1705 BCE. 1700 BCE (30.8%) 1660 BCE. 1655 BCE (11.2%) 1635 BCE	1760 BCE (95.4%) 1610 BCE	-1685
GV-IVc TS	seeds	GV-1725-23.1	KIA-32365	3380±30	3623	1730 BCE (10.5%) 1715 BCE. 1695 BCE (57.7%) 1630 BCE	1750 BCE (95.4%) 1610 BCE	-1674
GV-IVc TS	charcoal	GV-1597-25.1	KIA-32357	3370±40	3613	1740BCE (12.9%) 1710 BCE. 1700BCE (55.3%) 1610 BCE	1750 BCE (95.4%) 1530 BCE	-1664
GV-IVc TS	seeds	GV-1698-23.1	KIA-40425	3360±35	3603	1730 BCE (5.4 %) 1710 BCE. 1700 BCE (62.8%) 1610 BCE	1750 BCE (81.3%) 1600 BCE. 1590 BCE (14.1%) 1530 BCE	-1654
GV-IVc TSM	charcoal	GV-3082-25.1	KIA-37603	3395±30	3639	1740 BCE (62.5%) 1660 BCE. 1650 BCE (5.7%) 1640 BCE	1770 BCE (95.4%) 1610 BCE	-1690

PUNTA DE LOS GAVILANES SITE RADIOCARBON DATINGS. * M. Stuiver. PJ. Reimer. R. Reimer: ^{14}C Calibration Program. v.7.1. 2018. ** Few sample. * Low quality sample**
