# Supplementary material for: Revealing Ancient Wheat Phylogenetic Diversity: Machine Learning and Logistic Regression Identify Triticum sphaerococcum in Bronze Age Iberia
Source: Genes (Basel). 2025 Dec 9;16(12):1477. doi: 10.3390/genes16121477 (PMC12733154; doi:10.3390/genes16121477)
Supplement: Supplementary file 1 [file genes-16-01477-s001.zip › Supplementary Table S2.pdf]

Supplementary Table S2. Comparative morphometric analysis of modern reference and archaeobotanical wheat (*Triticum* spp.) caryopses from Gavilanes and Almizaraque archaeological sites. Measurements include length (L), breadth (B), and depth (D) in millimeters, with derived dimensional ratios (L/B, L/D, B/D) and breadth-to-length percentage (100*B/L). Modern reference specimens comprise experimentally carbonized seeds processed under controlled laboratory conditions to simulate archaeological preservation, alongside desiccated seed collections. Archaeological specimens were taxonomically assigned using parallel Random Forest and Logistic Regression algorithms trained on morphometric data from modern reference collections, with assignments accepted only when both models achieved probability scores >0.7. Standard deviations (sd) are provided in italics to the right of mean values.

		Length		Breadth		Depth		L/B		L/D		B/D		100*(b/L)	
Taxon	Seeds	mean	*sd*	mean	*sd*	mean	*sd*	mean	*sd*	mean	*sd*	mean	*sd*	mean	*sd*
Triticum aestivum L. subsp. *aestivum*, modern desiccated seeds	131	6.30	*0.90*	3.41	*0.44*	3.06	*0.39*	1.85	*0.19*	2.06	*0.17*	1.12	*0.09*	54.52	*6.23*
Triticum aestivum L. subsp. *aestivum*, experimentally carbonized modern seeds	50	5.53	*0.28*	3.70	*0.29*	3.00	*0.18*	1.50	*0.15*	1.85	*0.14*	1.23	*0.09*	67.09	*6.29*
Gavilanes, archaeobotanical	6	5.43	*0.34*	3.20	*0.28*	3.03	*0.27*	1.70	*0.09*	1.80	*0.07*	1.06	*0.06*	58.88	*3.08*
Almizaraque, archaeobotanical	44	4.78	*0.71*	2.65	*0.58*	2.49	*0.38*	1.83	*0.14*	1.93	*0.17*	1.06	*0.16*	55.01	*4.59*
Triticum aestivum subsp. *compactum* (Host) H.Messik., modern desiccated seeds	73	5.89	*0.53*	3.02	*0.29*	2.69	*0.34*	1.96	*0.19*	2.21	*0.23*	1.14	*0.13*	51.52	*5.14*
Triticum aestivum subsp. *compactum* (Host) H.Messik., experimentally carbonized modern seeds	50	5.02	*0.22*	3.11	*0.14*	2.48	*0.17*	1.62	*0.11*	2.03	*0.18*	1.26	*0.09*	62.02	*4.28*
Gavilanes, archaeobotanical	7	4.97	*0.66*	2.74	*0.29*	2.41	*0.56*	1.82	*0.20*	2.15	*0.58*	1.18	*0.21*	55.59	*6.05*
Almizaraque, archaeobotanical	67	4.87	*0.51*	2.82	*0.35*	2.43	*0.32*	1.74	*0.13*	2.02	*0.19*	1.16	*0.11*	57.89	*4.51*
Triticum aestivum var. *antiquorum* (Heer) H.Messik., archaeobotanical taxon	38	4.27	*0.78*	3.19	*0.46*	2.63	*0.44*	1.34	*0.17*	1.63	*0.18*	1.22	*0.11*	75.58	*8.64*
Gavilanes, archaeobotanical	7	4.20	*0*	2.73	*0*	2.70	*0*	1.54	*0*	1.55	*0*	1.01	*0*	65.54	*0*
Almizaraque, archaeobotanical	1	4.40	*0*	2.10	*0*	2.20	*0*	2.10	*0*	2.00	*0*	0.95	*0*	47.73	*0*
Triticum sphaerococcum subsp. *antiquorum* N.P.Gonch., modern desiccated seeds	59	4.71	*0.36*	2.54	*0.40*	2.81	*0.34*	1.88	*0.23*	1.69	*0.19*	0.91	*0.10*	53.89	*6.22*
Gavilanes, archaeobotanical	9	4.37	*0.62*	2.67	*0.28*	2.74	*0.21*	1.63	*0.15*	1.59	*0.17*	0.97	*0.09*	61.20	*6.25*
Almizaraque, archaeobotanical	50	4.62	*0.38*	2.57	*0.33*	2.64	*0.33*	1.81	*0.13*	1.76	*0.11*	0.98	*0.06*	55.53	*3.93*
Triticum sphaerococcum Percival subsp. *sphaerococcum* (*T. aestivum* subsp. *sphaerococcum* (Percival) Mac Key), modern desiccated seeds	58	5.04	*0.39*	3.43	*0.26*	3.15	*0.21*	1.47	*0.10*	1.61	*0.12*	1.09	*0.08*	68.13	*4.69*
Triticum sphaerococcum (Percival) Mac Key subsp. *sphaerococcum*, experimentally carbonized seeds	50	5.00	*0.31*	3.90	*0.35*	3.32	*0.29*	1.29	*0.10*	1.52	*0.13*	1.18	*0.12*	77.99	*5.92*
Gavilanes, archaeobotanical	28	4.88	*0.49*	3.33	*0.25*	3.18	*0.20*	1.47	*0.05*	1.54	*0.10*	1.05	*0.05*	68.65	*1.85*
Almizaraque, archaeobotanical	162	5.06	*0.30*	3.53	*0.21*	3.27	*0.24*	1.44	*0.10*	1.55	*0.12*	1.09	*0.08*	70.02	*4.82*
Triticum turgidum subsp. *parvicoccum* Kislev, archaeobotanical taxon	17	4.75	*0.57*	2.92	*0.36*	2.54	*0.35*	1.64	*0.21*	1.88	*0.23*	1.15	*0.04*	61.89	*7.07*
Gavilanes, archaeobotanical	7	4.61	*0.60*	2.81	*0.35*	2.61	*0.34*	1.64	*0.13*	1.78	*0.15*	1.08	*0.04*	61.01	*5.86*
Almizaraque, archaeobotanical	80	4.50	*0.30*	2.36	*0.19*	2.30	*0.28*	1.92	*0.13*	1.97	*0.19*	1.03	*0.08*	52.42	*3.42*
Triticum turgidum subsp. *carthlicum* (Nevski) Á.Löve & D.Löve, modern desiccated seeds	90	6.14	*0.65*	2.64	*0.37*	2.69	*0.31*	2.36	*0.30*	2.30	*0.25*	0.98	*0.08*	43.08	*5.11*

		Length		Breadth		Depth		L/B		L/D		B/D		100*(b/L)	
Taxon	Seeds	mean	*sd*	mean	*sd*	mean	*sd*	mean	*sd*	mean	*sd*	mean	*sd*	mean	*sd*
Almizaraque, archaeobotanical	10	5.30	*0.48*	2.60	*0.32*	2.57	*0.31*	2.05	*0.10*	2.07	*0.08*	1.01	*0.02*	48.93	*2.29*
Triticum aestivum subsp. *macha* (Dekapr. & Menabde) Mac Key, modern desiccated seeds	90	7.40	*0.57*	2.87	*0.41*	2.43	*0.32*	2.63	*0.36*	3.09	*0.34*	1.19	*0.16*	38.79	*5.24*
Gavilanes, archaeobotanical	1	4.80	*0.23*	2.30	*0.17*	1.40	*0.27*	2.09	*0.07*	3.43	*0.13*	1.64	*0.07*	47.92	*2.65*

Note: Modern reference taxa identified in the analysis include common wheat (*T. aestivum* subsp. *aestivum*), club wheat (*T. aestivum* subsp. *compactum*), Indian dwarf wheat (*T. sphaerococcum* subsp. *antiquorum* and subsp. *sphaerococcum*), Georgian wheat (*T. turgidum* subsp. *carthlicum*), and Georgian mountain wheat (*T. aestivum* subsp. *macha*). Samples of the archaeobotanical taxa *T. aestivum* var. *antiquorum*, and *T. turgidum* subsp. *parvicoccum* were also included in the training set. Zero standard deviations indicate single-specimen identifications. The integration of machine learning taxonomic assignment with traditional morphometric description enables robust identification of archaeological cereal remains while accounting for taphonomic alterations inherent in the archaeobotanical record. This approach facilitates comparative analysis between prehistoric agricultural assemblages and modern wheat diversity, contributing to understanding of crop evolution and ancient farming practices in the Iberian Peninsula.
