# Supplementary material for: Revealing Ancient Wheat Phylogenetic Diversity: Machine Learning and Logistic Regression Identify Triticum sphaerococcum in Bronze Age Iberia
Source: Genes (Basel). 2025 Dec 9;16(12):1477. doi: 10.3390/genes16121477 (PMC12733154; doi:10.3390/genes16121477)
Supplement: Supplementary file 1 [file genes-16-01477-s001.zip › Supplementary Table S3.pdf]

Supplementary Table S3. Synopsis of Nomenclature and Differential Characters for Archaeological Remains and modern taxa of Free-Threshing Domesticated Wheats[1].

Characters	*T. turgidum* subsp. *turgidum* (Rivet wheat)	*T. turgidum* subsp. *durum* (Desf.) Husn. (Durum wheat)	*T. turgidum* subsp. *carthlicum* (Nevski) Á. Löve & D. Löve (Persian wheat)	*T turgidum* subsp. *turanicum* (Jakubz.) Á. Löve & D. Löve (Khorassan wheat)	*T. turgidum* subsp. *polonicum* (L.) Thell. (Polish wheat)	*T. turgidum* subsp. *parvicoccum* Kislev (Small-Durum wheat)*	*T. aestivum* (subsp. *compactum*) var. *antiquorum* (Heer) H. Messik.*	*T. sphaerococcum* subsp. *antiquorum* N.P.Gonch.	*T. sphaerococcum* subsp. *sphaerococcum* (Percival) Mac Key	*T. aestivum* L. subsp. *aestivum*
Ploidy	Tetraploid	Tetraploid	Tetraploid	Tetraploid	Tetraploid	Tetraploid	Hexaploid	Hexaploid	Hexaploid	Hexaploid
Culm upper internode consistence	Solid	Solid or wall thick	Hollow	Solid or hollow	Solid	Solid	Hollow	Hollow	Hollow	Hollow, rarely solid
Spike shape	Dense, cylindrical	Dense, compact	Loose, elongated	Loose, elongated	Very long, lax	Compact, short	Dense, compact	Dense, short	Dense, short	Dense or lax,
Spike length (excluding awns)	7-11.5 cm	4-11 cm	9 cm	10-11.5 cm	(7)10-16 cm	>3.5 cm	4 cm	4-6 cm	4-6 cm	6-18 cm
Zigzag rachises	Lacking	ND	ND	ND	ND	Present	Present	ND	ND	Lacking
Shape of rachis node	ND	ND	ND	Often with a conspicuous rounded lump beneath each glume insertion	Often with a conspicuous rounded lump beneath each glume insertion	Often with a conspicuous rounded lump beneath each glume insertion	With either no lumps or weakly developed lower halves of lumps	With either no lumps or weakly developed lower halves of lumps	With either no lumps or weakly developed lower halves of lumps	With either no lumps or weakly developed lower halves of lumps
Shape of rachis internode	Flattened, narrow and wedge-shaped	Flattened, narrow and wedge-shaped	Narrow	Narrow and wedge-shaped	Flattened, narrow and wedge-shaped	Forming straight-sided trapeziums	Conspicuously shield-shaped, with a strongly curved widening of the upper third	ND	ND	Conspicuously shield-shaped, with a strongly curved widening of the upper third
Rachis hairs along the margins and frontal tuft at the base of each spikelet	White hairs, and a tuft of 1–2-mm-long white hairs	Long, and a tuft of hairs	Long, and a tuft of hairs	Few or no rachis hairs along the margins, generally lacks the frontal tuft of hairs	Hairs, and a tuft of hairs 2–2.5 mm long	Long, and a tuft of hairs 2 mm long	ND	Very short white hairs, also at the base of each spikelet	Very short white hairs, also at the base of each spikelet	Short hairs, also at the base of each spikelet
Rachis fragility	Semi-fragile	Non-fragile	Semi-fragile	Semi-fragile	Semi-fragile	Fragile	Tough	Tough	Tough	Tough
Spikelet density	High	High	Medium	Medium	Medium	Medium to high	High	High	High	Medium
Supernumerary spikelet spikes	Sporadic	Sporadic	ND	Sporadic	ND	ND	ND	Not	Not	Not
Spikelet length	10-13 mm	10-15 mm	ND	15-17 mm	30-40 mm	10-11 mm	6-10 mm	10 mm	10 mm	10-15 mm
Spikelet florets	4-7	5-7	2-4	2-4	4-5	2	3-5	6-7	6-7	4-9

Characters	*T. turgidum* subsp. *turgidum* (Rivet wheat)	*T. turgidum* subsp. *durum* (Desf.) Husn. (Durum wheat)	*T. turgidum* subsp. *carthlicum* (Nevski) Á. Löve & D. Löve (Persian wheat)	*T turgidum* subsp. *turanicum* (Jakubz.) Á. Löve & D. Löve (Khorassan wheat)	*T. turgidum* subsp. *polonicum* (L.) Thell. (Polish wheat)	*T. turgidum* subsp. *parvicoccum* Kislev (Small-Durum wheat)*	*T. aestivum* (subsp. *compactum*) var. *antiquorum* (Heer) H. Messik.*	*T. sphaerococcum* subsp. *antiquorum* N.P.Gonch.	*T. sphaerococcum* subsp. *sphaerococcum* (Percival) Mac Key	*T. aestivum* L. subsp. *aestivum*
Glume shape	Broad, rounded	Narrow, elongated	Intermediate	Intermediate	Long, narrow	Short, broad	Broad, rounded	Inflated	Inflated	Oblong, asymmetrical
Glume length	8-11 mm	8-12 mm	8-12 mm	12-15 mm	20-40 mm	7-13 mm	7-8 mm	8-9 mm	8-9 mm	6-11 mm
Persistence of glume bases	Pieces of glume-base commonly survive attached to rachis node	Pieces of glume-base commonly survive attached to rachis node	Pieces of glume-base commonly survive attached to rachis node	Pieces of glume-base commonly survive attached to rachis node	Pieces of glume-base commonly survive attached to rachis node	Pieces of glume-base commonly survive attached to rachis node	Glume-bases deciduous	Glume-bases deciduous	Glume-bases deciduous	Glume-bases deciduous
Angle between the glume and the rachis	Less than 45º	Less than 45º	Less than 45º	Less than 45º	Less than 45º	Less than 45º	More than 45º	More than 45º	More than 45º	More than 45º
Keel of the glume	Prominent keel of the glume running from the base to the tip	Prominent keel of the glume running from the base to the tip	Prominent keel of the glume running from the base to the tip	Prominent keel of the glume running from the base to the tip	Not prominent	Prominent keel of the glume running from the base to the tip	Prominent only in the upper half	Prominent only in the upper half	Prominent only in the upper half	Prominent only in the upper half
Lemma awn	Awnless, short or long-awned, (7)10-19 cm	Long-awned, (4)20-23 cm	Short-awned, 8-11 cm	Medium-awned, 14-16 cm	Medium and prominent, 7-15 cm	Awnless	Awnless	Awnless to short, 1.5-2 cm	Awnless to short, 1.5-2 cm	Awnless or 10-15 cm
Kernel color	Red or white	Amber (hard, vitreous)	Red or rarely white	Red or white	Red or white	ND	ND	Red or white	Red or white	White, yellow or red
Kernel shape	Rounded, plump	Slender	Intermediate	Intermediate	Elongated	Ovoid, ellipsoidal or rounded	Bluntly rounded	Distinctly shorter and rounder	Distinctly shorter and rounder	Generally plump
Kernel dimensions	Intermediate, mean 7.8 mm, ± 0.51	Long, mean 8.3 mm, ± 0.24	Intermediate, mean 6.1 mm, ± 0.65	Large, 10.5-12 mm	Large, mean 11.9 mm, ± 0.87	Small, mean 4.8 mm, ± 0.57	Small, mean 4.3 mm, ± 0.78	Small, mean 4.7 mm, ± 0.36	Small, mean 5 mm, ± 0.39	Small to long, Small, mean 6.3 mm, ± 0.9
Adaptation	Mediterranean and Temperate regions	Mediterranean regions	Caucasus, Middle East	Central Asia	Mediterranean, Central Asia	Middle East	Mediterranean and Temperate regions	Central Asia	Central Asia	Mediterranean and Temperate regions

[1] This table provides a comprehensive overview of the naming conventions and distinguishing features used to identify archaeological remains of free-threshing domesticated wheat. It highlights the key characteristics that differentiate various types of wheat, offering a clear and concise reference for archaeologists and researchers studying ancient agricultural practices. * Only known in carbonized state. ND = No data available. **References**: [25, 26, 35, 37, 40, 41, 43, 46, 47, 49, 59, 63, 64, 66]
